# From Evidence to Practice: The Growing Role of Angiography-Derived Physiology

**DOI:** 10.3390/jcm14228219

**Published:** 2025-11-20

**Authors:** Daniel K. Amponsah, William F. Fearon

**Affiliations:** Division of Cardiovascular Medicine and Cardiovascular Institute, Stanford, CA and VA Palo Alto Health Care System, Stanford University School of Medicine, Palo Alto, CA 94305, USA

**Keywords:** angiography-derived physiology, fractional flow reserve, coronary artery disease

## Abstract

Angiography-derived physiology (ADP) has emerged as a validated, wire-free method for the functional assessment of coronary artery disease (CAD). By avoiding pressure-wire instrumentation and hyperemic agents, ADP reduces procedure time, radiation exposure, and cost, while maintaining strong diagnostic performance with invasive physiology. These platforms include FFRangio (CathWorks), QFR (Medis Medical Imaging), and vFFR (Pie Medical Imaging), which have undergone extensive validation and are FDA approved for use. Randomized trials, predominantly with QFR, thus far demonstrate improved outcomes of ADP-guided strategies compared with angiography alone, whereas non-inferiority to wire-based FFR guidance has not yet been established. As clinical trials continue, thoughtful integration into routine practice requires careful image acquisition, platform-specific training, and awareness of limitations. In particular, validation remains incomplete in complex subsets such as left main disease, bifurcations, and bypass grafts, though evidence is growing in the application in acute coronary syndromes, post-PCI prognostication, and surgical planning. As ongoing studies mature and ADP technology evolves, these tools are poised to reshape physiologic assessment, streamline catheterization laboratory workflow, and become integral to contemporary PCI planning and optimization. This review summarizes current evidence, clinical applications, limitations, integration into the catheterization lab, and future directions of ADP.

## 1. Introduction

**Figure 1:**
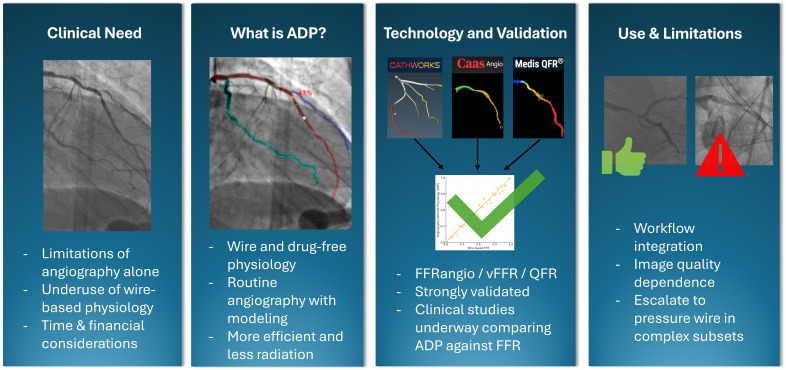
Overview of angiography-derived physiology outlining the clinical need, core principles, validation across major platforms, and practical use considerations.

Coronary angiography remains the gold standard for the evaluation of coronary artery disease (CAD), allowing for the precise diagnosis and treatment of obstructive lesions [1,2]. Over time, it became apparent that revascularization of severely obstructive disease provided benefit, but the magnitude of benefit was less clear in patients with more moderate coronary disease. To address this gap, physiological assessment of coronary arteries has become an essential tool to reliably distinguish moderate lesions that can be safely deferred from those that may benefit from revascularization [3,4,5]. Although this strategy resulted in fewer stent implantations, it was associated with improved outcomes, ultimately leading to the incorporation of wire-based physiology into clinical guidelines [1,3]. Alongside this development, efforts were made to evaluate moderate CAD without the need for a pressure wire, giving rise to angiography-derived physiology (ADP) [6]. This tool has seamlessly emerged as a promising advancement in coronary physiology, streamlining physiologic evaluation by eliminating the need for pressure-wire instrumentation and hyperemic medication, ultimately providing rapid and useful results. Furthermore, ADP technologies have been reported to shorten procedure time, reduce radiation exposure, and lower costs [7]. These technologies have been extensively validated against traditional pressure-wire-based physiology, leading to U.S. Food and Drug Administration (FDA) clearance of several ADP software platforms [6]. Building on this, randomized controlled trials have evaluated clinical outcomes with this technology versus angiography alone and versus wire-based physiology, with additional studies underway [6]. Despite the overall promising results of coronary physiology in the management of patients with CAD, adoption of coronary physiology in practice remains slow [8]. In this review, we aim to review ADP technologies, summarize the validation of each FDA approved ADP software, critically evaluate evidence derived from clinical trials, examine the clinical applicability of these technologies, and identify limitations and barriers while proposing practical solutions to overcome these barriers (Figure 1).

### 1.1. What Is Angiography-Derived Physiology

The evaluation of coronary physiology stems from our understanding of coronary flow reserve (CFR) and the capacity of the coronary vasculature to dynamically change when demand increases [9]. CFR became an early surrogate for the evaluation of coronary lesions with a low CFR, indicating a higher likelihood of flow-limiting disease [9]. However, CFR reflects the entire coronary circulation, being affected by both the epicardial vessels and the microvasculature. To minimize microvasculature contribution, maximal hyperemia was used, and fractional flow reserve (FFR) was developed as a pressure-based measurement that isolates epicardial vessels [10,11]. This was later adapted to an alternative, non-hyperemic pressure ratio (NHPR) technique that similarly assesses epicardial vessels, but under resting conditions [12].

**Figure 2:**
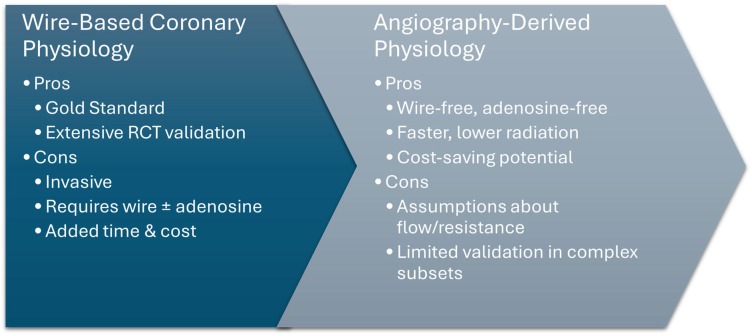
Wire-based physiology vs. angiography-derived physiology.

The development of ADP emerged from the recognition that cine-densitometric methods can measure absolute coronary blood flow, which in turn allows calculation of mean transit time of contrast during rest and hyperemia to derive CFR [13]. Leveraging this concept, computational fluid dynamics and similar equations were integrated with standard coronary angiography, enabling the creation of accurate 3D coronary reconstructions to evaluate changes in flow across a lesion [6] (Figure 2). This approach simulates blood flow and identifies physiologically significant lesions, comparable to pressure-wire evaluations and by combining the diameter and length of a lesion, epicardial resistance can be estimated. Performance of this technology requires meticulous image acquisition and although largely automated, training is still required to ensure cine suitability and reliable lesion selection with skills to edit as required for accurate assessment of lesions.

### 1.2. Angiography-Derived Physiology: Technologies and How They Differ

Several commercial platforms provide ADP, each with specific acquisition requirements, computational approaches, manual inputs, and final outputs (Table 1). The FDA has approved several ADP technologies, including quantitative flow ratio (QFR) by Medis Medical Imaging, FFRangio by CathWorks, and vessel FFR (vFFR) by Pie Medical CAAS Workstation [14,15,16]. Understanding the differences between platforms is essential for protocol design, staff training, and integration into the catheterization lab. For all technologies, two or more high-quality angiograms, with minimal vessel overlap and adequate opacification, are required to avoid hidden eccentric lesions on single views or vessel foreshortening, with all having a cut-off value of ≤ 0.80 for hemodynamic significance [6].

**Figure 3:**
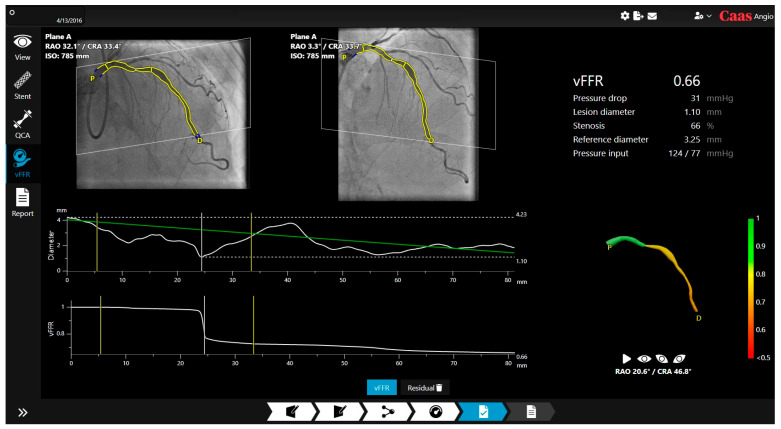
Example of vFFR output interface (reprinted with permission from Ref. [19]. Copyright 2025 Pie Medical Imaging).

vFFR was developed on the principle of the Navier–Stokes equations, which are fundamental partial differential equations to describe the motion of fluids [6] (Figure 3). This software requires two angiographic views at least 25 degrees apart and supports single vessel analysis [17]. It can generate a virtual FFR pullback and provide a reference diameter for sizing; with a recent update, residual vFFR estimates post-treatment physiology to predict procedural effectiveness [18]. The system also features co-registration of residual vFFR which does not have FDA clearance and is not currently available for clinical use in the United States.

**Figure 4:**
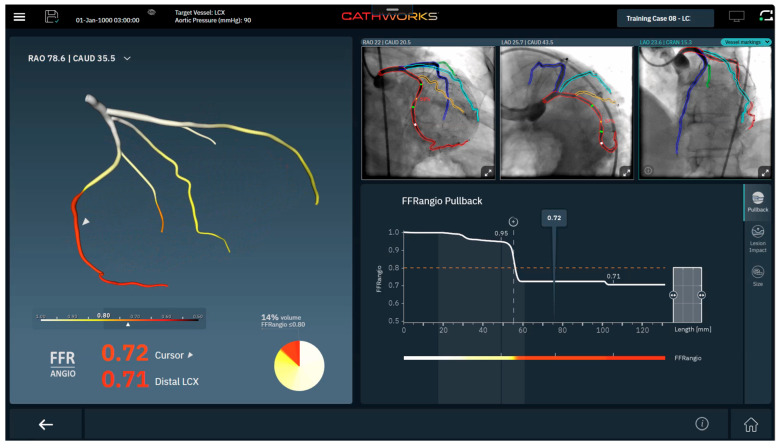
Example of FFRangio Output Interface (Reprinted with permission Ref. [21]. Copyright 2025 CathWorks).

FFRangio uses a proprietary flow resistance analysis algorithm to conduct multivessel analysis [20] (Figure 4). This technology requires three angiographic views separated by at least 30 degrees, reporting FFRangio values along the coronary tree. Recent software upgrades add (I) pullback analysis to determine lesion-specific contributions to flow limitation, (ii) a lesion impact tool to estimate relative change in flow with stent placement, and (iii) vessel-sizing to estimate lesion length and vessel size [21].

**Figure 5:**
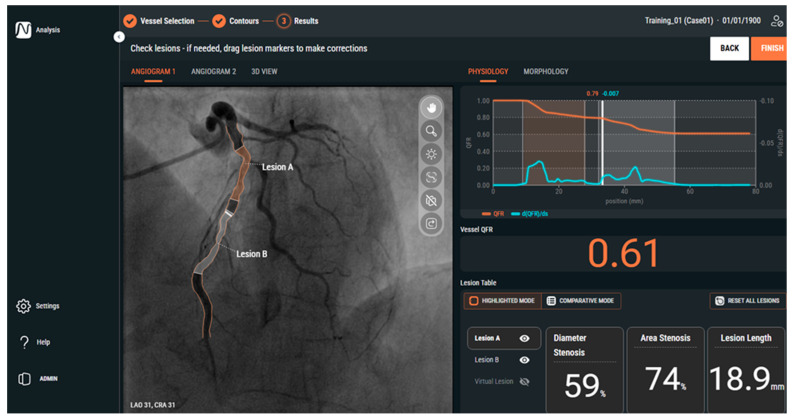
Example of QFR output interface (reprinted with permission from Ref. [25]. Copyright 2025 Medis Medical Imaging).

QFR uses a similar method to vFFR, with the simplified Navier–Stokes equation similarly requiring two orthogonal angiograms separated by at least 25 degrees [22] (Figure 5). This platform identifies functionally significant lesions, with additional features that determine stent size and length for optimal stenting and post-percutaneous coronary intervention (PCI) QFR prediction [23]. The additional features have not received FDA clearance currently and are still under ongoing validation. QFR is also in the development of an angiography-derived index of microcirculatory resistance called IMRangio which is currently under investigation [24].

### 1.3. Angiography-Derived Physiology: Validation

ADP has been extensively validated against wire-based coronary physiology across multiple prospective cohorts and multicenter studies. These studies have consistently demonstrated strong diagnostic agreement, supporting lesion-level decision making (Table 2).

FFRangio’s earliest validation study was a single-center study of 80 patients and 101 lesions in patients with stable, moderate CAD, which demonstrated good diagnostic performance with a diagnostic accuracy of 94%, specificity of 98%, and sensitivity of 88% [26]. This was followed by the FFRangio Accuracy versus Standard FFR (FAST-FFR) study, a multicenter, blinded prospective study where FFRangio was validated against wire-based FFR [20]. The investigators demonstrated excellent diagnostic concordance of FFRangio to wire-based FFR with a diagnostic accuracy of 92%, sensitivity of 94%, and specificity of 91% to detect an FFR ≤ 0.80. Lastly, a pooled analysis of five studies evaluating FFRangio compared to wire-based FFR with a total of 700 lesions revealed a diagnostic accuracy of 93%, sensitivity of 91%, and specificity of 94% [27].

QFR has undergone extensive validation, first entering prospective evaluation with the FAVOR (Functional Assessment by Various Flow Reconstructions) pilot study, which was a prospective, multicenter trial with stable coronary artery disease and moderate lesions [22]. Investigators demonstrated a diagnostic accuracy of 80%, sensitivity of 74%, specificity of 91%, positive predictive value (PPV) of 83%, and negative predictive value (NPV) of 86% to detect an FFR ≤ 0.80. This was followed by the FAVOR II China study which revealed concordance with wire-based FFR, demonstrating diagnostic accuracy of 92.7%, sensitivity of 94.6%, specificity of 91.7%, PPV of 85.5%, and NPV of 97.0% [28]. Published in near parallel, the FAVOR II Europe-Japan study showed a diagnostic accuracy of 86.9%, sensitivity of 86.5%, specificity of 86.9%, PPV of 76.3%, and NPV of 93.0% [29]. This was followed by the WIFI II study that showed a sensitivity of 77% and specificity of 86% [30]. Lastly, a pooled analysis of 16 prospective studies evaluating diagnostic accuracy in QFR against wire-based FFR with 819 patients and 969 vessels showed a sensitivity of 84%, specificity of 88%, PPV of 80%, and NPV of 95% [31].

vFFR prospective evaluation includes the FAST study which was a single-center study that confirmed excellent accuracy with an AUC of 0.93 to detect flow-limiting lesions [17]. The FAST EXTEND study showed strong concordance with a diagnostic accuracy of 88%, sensitivity of 75%, specificity of 94%, PPV of 84%, and NPV of 89% [32]. To follow up this study, the FAST II study, a multicenter prospective study including 334 patients, confirmed good diagnostic accuracy with a sensitivity of 81%, specificity of 95%, PPV of 90%, and NPV of 90% [33].

With several platforms now validated, attention has shifted to demonstrate clinical utility in comparison to angiography-based decision making as well as wire-based FFR strategies. If these trials confirm clinical utility, ADP will be poised for inclusion in clinical guidelines.

### 1.4. Randomized Control Trials of Angiography Derived Coronary Physiology

With the validation of ADP against wire-based FFR, ADP now must demonstrate whether it can guide revascularization and match or exceed outcomes achieved with angiography alone or wire-based FFR. Multiple trials have been completed or are underway to test whether an ADP-guided strategy can serve as a standalone approach Table 3.

QFR has undergone several international randomized control trials to determine this system’s utility compared to angiography alone and wire-based FFR. First, the FAVOR III China (Functional Diagnostic Accuracy of Quantitative Flow Ratio in Online Assessment of Coronary Stenosis III China) study, a multicenter, randomized clinical trial comparing QFR to angiography alone for clinical decision making to guide revascularization [34]. Patients with acute and chronic coronary syndromes and intermediate lesions (50–90%) were included. The primary endpoint of the trial was major adverse cardiac events (MACE), defined as the composite of death from any cause, myocardial infarction (MI), or ischemia-driven revascularization at one year. In total, 3825 patients were included, 1913 in the QFR-guided arm and 1912 in the angiography-guided arm. For patients randomized to the QFR-guided strategy, PCI was indicated if QFR ≤ 0.80. At one year, patients randomized to QFR had lower MACE compared to angiography alone (HR 0.65; 95% CI 0.51–0.83; *p* = 0.0004). This benefit was primarily driven by a reduction in MI and ischemia-driven revascularization. The study also revealed that patients in the QFR arm had a reduction in PCI by 10% due to treatment deferral. Moreover, the 2-year follow-up analysis showed that QFR-guided revascularization strategy continued to have better outcomes (HR 0.66, 95% CI: 0.54–0.81; *p* < 0.0001) [35]. This is the first large, multicenter study demonstrating the clinical utility of ADP technology assessment in guiding revascularization.

The subsequent study, named FAVOR III Europe (Functional Diagnostic Accuracy of Quantitative Flow Ratio in Online Assessment of Coronary Stenosis III Europe), compared clinical outcomes of QFR-guided versus wire-based FFR-guided revascularization in patients with intermediate coronary stenosis [36]. This trial was a multicenter, randomized non-inferiority trial enrolling 2000 patients with a primary composite endpoint of all-cause death, MI or unplanned revascularization at 1 year. At 12 months, 6.7% of patients in the QFR arm and 4.2% in the FFR arm met the primary endpoint with a hazard ratio of 1.63 (95% CI 1.11–2.41). The trial failed to demonstrate noninferiority of QFR to FFR for guiding revascularization in intermediate coronary lesions. Notably, QFR tended to label more lesions as physiologically significant compared to FFR, which led to more stent implantations (823 vs. 650). Although one may think peri-procedural MI being included in MI within the composite outcome may explain the difference seen in MACE, since more patients underwent PCI, there remained a significant difference in spontaneous MI during follow-up in QFR compared to FFR (2.7% vs. 1.3%) [37]. A subsequent substudy revealed that even in patients deferred based on QFR or FFR, event rates were higher in the QFR arm [38], suggesting that QFR may not have only led to unnecessary stenting but also may have left significant lesions unstented.

**Figure 6:**
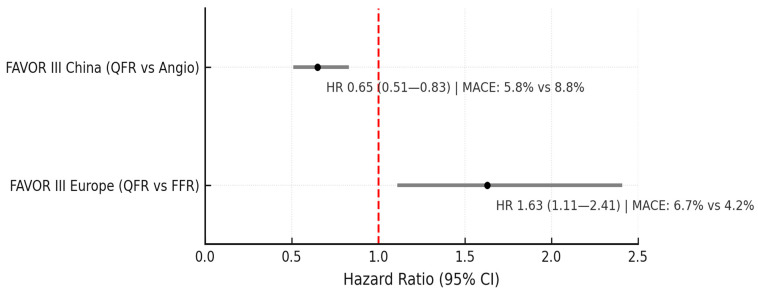
Forest plot of FAVOR III CHINA and FAVOR III Europe results; the red dashed line denotes the null effect (HR = 1.0).

QFR outperformed angiography-guided PCI but was inferior to wire-based FFR (Figure 6). Although QFR shows good accuracy compared to wire-based FFR based on validation studies, the findings from FAVOR III Europe indicate that pressure-wire FFR remains preferred in many settings compared to QFR-guided revascularization.

The Prospective Randomized Trial Evaluating Clinical Outcomes of Angiography-based Fractional Flow Reserve Guidance Versus Wire-based Fractional Flow, PROVISION study was recently presented as the first prospective, randomized non-inferiority trial comparing FFRangio to wire-based FFR in patients with stable CAD [39]. This study enrolled 401 patients with intermediate CAD with a primary endpoint of noninferiority in the revascularization rate guided by FFRangio versus the revascularization rate guided by FFR. Secondary endpoints evaluated MACE as a composite of death from any cause, nonfatal MI, or unplanned revascularization at 1 year, medical costs associated with the treatments, and radiation exposure. The investigators reported that FFRangio met non-inferiority compared to FFR for revascularization rate. At one-year follow-up, the investigators also demonstrated that 9.9% of patients in the FFRangio arm compared to 12.6% in the FFR arm suffered from death, nonfatal MI, or unplanned revascularization with a hazard ratio of 0.80 (95% CI 0.42–1.51, *p* = 0.489). There was a significant reduction in overall cost of $374–$400 and a reduction in radiation exposure in the FFRangio arm compared to wire-based FFR. FFRangio has recently completed enrollment for the Advancing Cath Lab Results with FFRangio Coronary Physiology Assessment (ALL-RISE-NCT05893498) which is an international prospective, randomized, multi-center, controlled post-market study [40]. The ALL-RISE study also aims to compare FFRangio-guided treatment to pressure wire-guided treatment in patients with CAD being considered for PCI, evaluating MACE at one year.

vFFR has completed enrollment for the FAST III trial (NCT04931771); this is a randomized trial comparing vFFR versus FFR-guided revascularization with MACE at one year as the primary endpoint [41]. Similarly, the LIPSIASTRATEGY trial (NCT03497637) remains ongoing, testing a similar vFFR versus FFR-guided strategy [42].

These trials remain critical to defining the clinical utility of ADP in guiding evidence-based integration into practice. Their outcomes will clarify where ADP can safely substitute, complement, or yield to wire-based FFR in the management of CAD, directly informing future guidelines recommendations. By clarifying strengths and limitations, they will provide practical guidance for workflow, training, and reimbursements. Because each method for performing ADP is distinct and unique, it is critical that each technique be tested independently in clinical outcomes studies to determine its utility.

### 1.5. Special Lesions

ADP has primarily been validated in moderate CAD with recognized limitations in select patient and lesion subsets. While no prospective randomized trials exist in complex lesions, observational and subgroup studies have explored its potential use in this population Table 4.

### 1.6. Acute Coronary Syndrome

Application of ADP in the setting of acute coronary syndrome (ACS), particularly for non-culprit lesion assessment, has drawn considerable interest. FFR-guided revascularization of non-culprit lesions in ST Elevation Myocardial Infarction (STEMI) has shown benefit [43]; however, there is concern that global microvascular dysfunction, which might occur at the time of a large STEMI, could transiently impact the FFR measurement in a non-culprit vessel. In theory, ADP should not be affected by this transient microvascular dysfunction. For QFR, several studies have demonstrated the feasibility of performing QFR in this population using angiograms obtained during the index event [44,45]. Despite concerns for altered coronary physiology in the acute phase, QFR continued to show strong diagnostic performance against wire-based FFR (AUC 0.887) with practical triage cutoffs (<0.75 to treat and >0.92 to defer) [44,46]. Similarly, acute phased QFR was evaluated in intermediate non-culprit lesions in patients with STEMI, showing 84% agreement between acute QFR and staged FFR and 74% agreement between acute QFR and staged iFR, supporting its reliability in the acute phase evaluation using QFR [45].

For vFFR, the FAST-STAGED study evaluated the change in index vFFR of a non-culprit lesion compared to staged vFFR values in STEMI patients to confirm the accuracy compared to the acute phase [47]. This study showed a diagnostic accuracy of 93.5%, sensitivity of 96.4%, specificity of 88.9%, PPV of 93.1%, and NPV of 94.1% [47]. The FAST STEMI II study was recently presented at TCT 2024, which revealed modest diagnostic performance between vFFR versus FFR-guided complete revascularization in patients presenting with STEMI with a diagnostic accuracy of 71.8%, sensitivity of 76.5%, specificity of 69.7%, PPV of 53.1%, and 86.9% [48]. Discordance between vFFR and FFR was most often seen in patients with microvascular dysfunction.

For FFRangio, a study was conducted to evaluate the diagnostic performance of intermediate lesions in patients presenting with Non-ST Elevation Myocardial Infarction [49]. This study showed high diagnostic accuracy against wire-based FFR in this patient population with a diagnostic accuracy of 96.7%, sensitivity of 95.5%, and specificity of 97.4% [49]. This suggests that the technology can be applied reliably in ACS when physiology-guided decision making is desired. It will be important to determine if ADP in the non-culprit vessel at the time of ACS is able to predict FFR/NHPR months later, once the microvascular dysfunction and hemodynamic changes have been resolved.

### 1.7. Left Main Coronary Artery Disease

Accurate assessment of left main (LM) coronary disease remains a clinical priority given the prognostic weight of revascularization decisions. Data for QFR are emerging within patients with LM lesions showing acceptable agreement with invasive FFR for intermediate LM lesions with a diagnostic accuracy of 90.7%, sensitivity of 88.1%, specificity of 92.3%, PPV of 88.1%, and NPV of 92.3% [50]. A similar study reported that QFR could identify functionally significant LM disease with reasonable accuracy, demonstrating a sensitivity of 85.4%, specificity of 69.6%, PPV of 85.5%, and NPV of 64% [51].

For vFFR, a study evaluating vFFR’s correlation to intravascular ultrasound (IVUS) in intermediate lesions showed that vFFR correlated strongly with IVUS-derived minimal lumen area (MLA), with a diagnostic accuracy of vFFR ≤0.80 in identifying lesions with MLA < 6.0 mm^2^, showing a sensitivity of 98% and specificity of 71.4% [52]. This reinforces its feasibility, although most investigators still advocate for IVUS as the definitive reference when LM disease is borderline or complex.

For FFRangio, evidence in LM lesions remains sparse. Early validation studies excluded patients with LM disease and aorto-ostial lesions with no LM-focused observational studies published. Until dedicated data are available, physiologic assessment of LM lesions should be conducted using wire-based FFR or IVUS.

### 1.8. CABG and Graft Patency

ADP has also been investigated in the context of surgical planning and graft patency. Among the platforms, QFR has the most advanced evidence. In a prospective study conducted in 22 patients (65 vessels) undergoing coronary artery bypass, a QFR > 0.80 was associated with an increased risk of graft occlusion (58.6% vs. 17.0%, *p* = 0.03) [53]. For pre-operative planning, a study showed that pre-operative LAD QFR was independently associated with internal mammary graft failure and adverse outcomes, showing a QFR > 0.80 associated with a higher rate of graft failure (31.4% versus 7.2%, *p* < 0.001) [54]. These data support the concept that competitive flow arises in non-physiologically significant lesions predisposing to graft failures.

In contrast, no dedicated graft outcomes studies have been published for FFRangio or vFFR. Both modalities have been validated extensively against invasive FFR in native coronary lesions, but their role in CABG target selection and long-term graft performance remains a research gap. This is particularly important given that randomized invasive FFR-guided CABG trials have failed to demonstrate clear improvements in clinical outcomes compared with angiography-guided strategies [55]. Whether ADP can add value beyond these invasive data warrants further investigation.

### 1.9. Bifurcation Lesions

Bifurcation lesions tend to also be challenging to manage from an angiographic-guided PCI standpoint, requiring careful planning and strategies when it comes to revascularization. vFFR, FAST-EXTEND, and FAST II validation studies included bifurcation subsets reporting strong correlation with wire-based FFR [32,33]. QFR has also been evaluated in bifurcation lesions in a substudy of FAVOR III CHINA which showed that post-PCI QFR in the main and side branches demonstrated that functionally incomplete revascularization was associated with higher 1-year adverse events (25.2% vs. 12.3%; *p* < 0.0001) [56]. ADP serves as a potentially useful guide in the evaluation of bifurcation lesions.

### 1.10. Diffuse Serial Disease

Several ADP software have developed methods to assess diffuse and serial lesions. QFR has shown good consistency mimicking pressure-wire pullback, allowing for function assessment of diffuse CAD vs. focal lesions [57]. FFRangio produces a whole tree physiology map, useful for identifying the ischemic segment in diffuse or serial lesions [21]. vFFR has a pressure drop function that allows for discrimination between serial lesions [18]. These features can be helpful in multivessel disease or staged procedural planning

### 1.11. Chronic Total Occlusion

Chronic total occlusion (CTO) PCI volume has grown with advances in percutaneous coronary techniques. In a retrospective study, lower post-PCI QFR values after CTO intervention were associated with worse outcomes [58]. Evidence for vFFR and FFRangio in CTOs remains limited, as pre-PCI assessment is not feasible in fully occluded lesions and thus excluded from trials. ADP may therefore have a role in post-PCI prognostication.

### 1.12. Aortic Stenosis

Physiologic assessment in aortic stenosis (AS) is of particular interest as AS typically alters coronary physiology through changes in flow hemodynamics and microvascular function. In a small prospective study, QFR showed good agreement with wire-based FFR in patients with severe AS, demonstrating a diagnostic accuracy of 84%, sensitivity of 73%, specificity of 91%, PPV of 84%, and a NPV of 84% [59]. In a separate post-transcatheter aortic valve implantation (TAVI) study, QFR again showed good agreement with wire-based FFR with a diagnostic accuracy of 83% [60]. Evidence using FFRangio and vFFR is limited in patients with AS. Overall, these findings suggest ADP may be a useful adjunct for peri-TAVI assessment of moderate lesions, though larger studies are needed. Just as in ACS, it will be important to see if pre-TAVI ADP values correlate more closely with FFR values months after TAVI, when the hemodynamic and microvascular changes from AS have resolved.

### 1.13. Microvascular Dysfunction

ADP primarily models epicardial pressure-flow relationships and its assumptions about microvascular resistance can be violated in the presence of significant microvascular dysfunction. As a result, epicardial indexes may appear normal despite ischemia driven by microvascular dysfunction or conversely may underestimate epicardial severity when microvascular resistance is elevated. To address this gap, QFR-derived IMRangio (an angiography-derived index of microcirculatory resistance) has been proposed and is under active investigation [24,61]. Conceptually, IMRangio combines angiographic flow estimates with pressure-drop modeling to approximate wire-based IMR, allowing microvascular assessment from routine cine runs. While initial studies are encouraging, IMRangio remains investigational and not FDA-cleared. CathWorks and other companies are developing their own ADP methods for assessing microvasculature.

### 1.14. Post-PCI Coronary Physiology

Multiple ADP platforms have evaluated the diagnostic accuracy and outcomes of post-PCI physiologic indices. In the prospective multicenter HAWKEYE study, a post-PCI QFR ≤ 0.89 predicted a 3-fold higher risk in vessel-oriented events at 2 years, supporting the role of QFR for post-PCI prognostication [62]. Additional retrospective studies have confirmed these findings, reporting consistent prognostic associations for post-PCI physiology [63]. vFFR has likewise been validated post-PCI, demonstrating good agreement against invasive FFR to detect a post-PCI < 0.90 [64]. Long-term follow-up studies further showed that lower post-PCI vFFR values were associated with higher target vessel failure at 5 years. [65]. By contrast, evidence for FFRangio in the post-PCI setting remains limited, with ongoing randomized trials aiming to address this gap. Taken together, post-PCI ADP may help identify patients with suboptimal physiology results who might benefit from further optimization.

### 1.15. Intravascular Imaging

Intravascular imaging (IVI) modalities such as IVUS and optical coherence tomography (OCT) have become integral to the contemporary invasive management of CAD [66]. Randomized and observational data suggest that combining wire-based FFR and IVI can improve post-PCI physiology and stent optimization and better identify high-risk lesions, prompting interest in whether ADP may offer similar complementary value [67,68,69]. For QFR, lower values have been associated with IVUS- and OCT-defined high-risk plaque features and greater stenosis severity, supporting concordance between anatomic and functional assessments [70,71,72,73]. For vFFR, studies likewise demonstrate associations between reduced vFFR and suboptimal OCT lumen metrics and high-risk characteristics [74,75]. Data linking FFRangio with IVI are more limited, although early work suggests reasonable agreement between FFRangio-derived vessel sizing and IVUS measurements [76]. Collectively, these studies indicate that ADP and IVI provide complementary information and that their combined use is mechanistically and practically appealing; however, robust prospective trials evaluating predefined integrated ADP + IVI strategies with clinical endpoints are still lacking.

Although the management of CAD can be complex, ADP shows promise across challenging subsets and populations from non-culprit lesion triage in patients with ACS, LM significance, graft patency predictions, bifurcation side branch physiology, diffuse or serial disease using physiological pullback or tree-mapping, post-PCI prognostication in CTO, peri-TAVI coronary assessment, microvascular dysfunction through IMRangio, and complementary use with IVI. While many of these applications remain early in validation, they highlight the versatility and future potential of ADP.

## 2. Limitations

Although ADP represents a major advancement in the functional assessment of CAD, several important limitations remain that must be acknowledged.

### 2.1. Image Quality

The accuracy of ADP is fundamentally dependent on high-quality angiography. All currently available platforms require projections with minimal vessel foreshortening and overlap, stable catheter engagement, minimal panning of the table, and adequate contrast opacification. In the real-world Cath lab environment, these conditions cannot always be obtained due to numerous reasons. Suboptimal angiographic projections, patient motion, respiratory artifact, or arrhythmias can all degrade computational accuracy. Failure rates due to poor image quality or operator inexperience have been reported in the range of ~10% in early registries [20,44,77]. These limitations extend across ADP platforms, as demonstrated in a recent comparative validation study of QFR, vFFR, caFFR, and μQFR, in which diagnostic accuracy was affected by image utility and lesion characteristics; notably, FFRangio was not included in this analysis [78]. Therefore, attention to image acquisition protocols and willingness to repeat suboptimal cine runs are crucial for reliable results.

### 2.2. Hemodynamic Assumptions

Another key limitation lies in the hemodynamic modeling and assumptions made in ADP development. QFR, FFRangio, and vFFR all assume stable coronary flow and use population-based models to estimate microvascular resistance. These assumptions may not hold true in conditions associated with dynamic or abnormal microvascular physiology, such as acute coronary syndromes, diabetes, severe left ventricular dysfunction, or hypertrophic cardiomyopathy. In these contexts, ADP can misclassify lesion severity because epicardial physiology does not fully reflect the downstream microvascular environment. However, as mentioned above, this may also be an advantage of ADP because it is not affected by transient microvascular dysfunction in the setting of STEMI or reversible microvascular dysfunction in the setting of severe AS.

### 2.3. Limited Validation in Special Subsets

The strongest validation of ADP has been in intermediate lesions of stable CAD. In more complex subsets like left main, bifurcations, CTO, bypass grafts, and diffuse disease, validation is less robust. In these settings, ADP may be useful as an adjunct but should not replace wire-based physiology or intravascular imaging.

### 2.4. Lack of Microvascular or Perfusion Information

Unlike positron emission tomography, cardiac magnetic resonance, or invasive indices such as CFR and index of microcirculatory resistance, ADP interrogates only the epicardial coronary tree. It does not account for microvascular dysfunction or provide information on myocardial perfusion. Consequently, patients with ischemia with non-obstructive coronary arteries or myocardial infarction with non-obstructive coronary arteries may have “normal” ADP values despite demonstrable ischemia. This limits the applicability of ADP in patients with microvascular angina or other non-epicardial causes of ischemia. Medis Medical Imaging, the developers of QFR, have introduced IMRangio, a software tool designed to estimate microvascular resistance, and other companies are developing their own indices; this application remains under active investigation.

In summary, the limitations of ADP span technical, physiologic, and clinical domains. They include dependence on angiographic quality, reliance on simplified hemodynamic assumptions, incomplete validation in complex lesion subsets, and lack of microvascular assessment. Recognition of these limitations is essential to guide appropriate integration of ADP into clinical practice and to identify areas for future research.

## 3. How to Integrate ADP into Daily Practice

**Figure 7:**
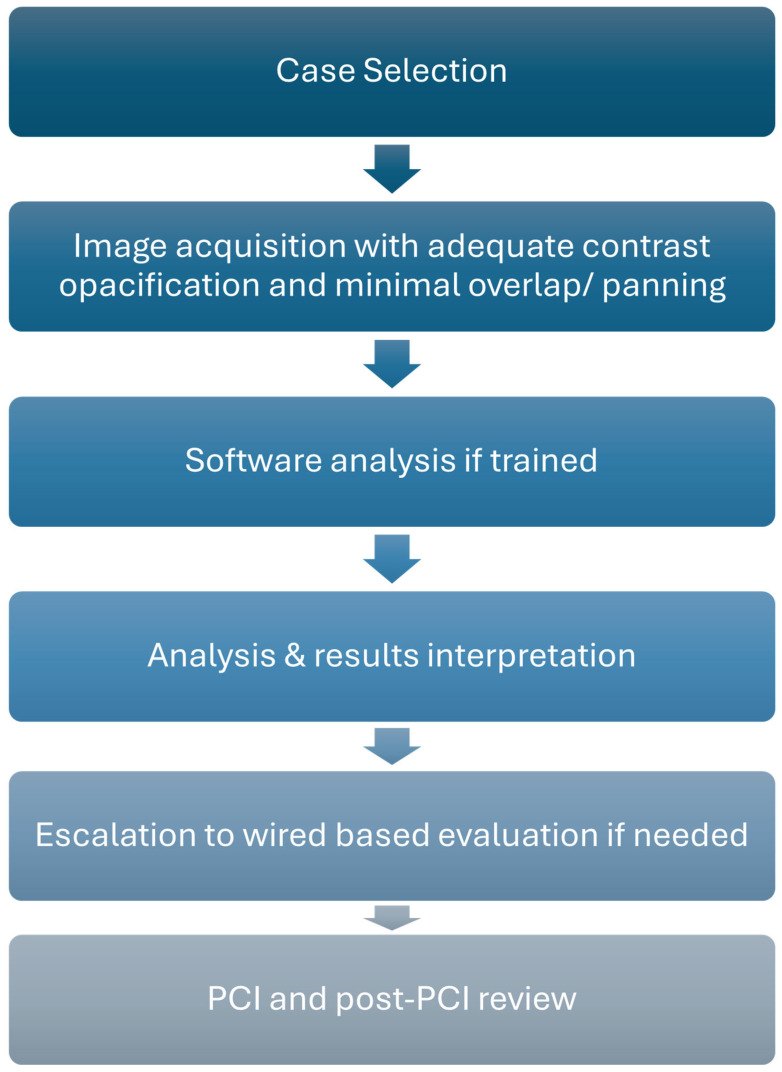
Integration workflow into daily practice.

Successful adoption of ADP into daily workflow requires deliberate attention to acquisition technique, training, workflow integration, and a structured framework for interpretation and quality review. Although these tools are increasingly automated, their accuracy and reliability remain dependent on thoughtful implementation. We propose a stepwise workflow from case selection through post hoc quality review that can be embedded into routine PCI practice (Figure 7).

### 3.1. Case Selection

At present, the strongest evidence base lies in the evaluation of intermediate lesions in stable CAD. There is increasing data supporting its use in ACS non-culprit assessment; however, evidence in left main, bifurcation, diffuse, and calcified disease remains under investigation. In such subsets, ADP may provide supportive information but should not be regarded as definitive and may warrant a default escalation to invasive physiology or intravascular imaging for further assessment.

### 3.2. Image Acquisition

The cornerstone of reliable ADP is high-quality angiography. Projections should minimize vessel foreshortening and overlap with operators able to adjust fluoroscopic angles according to patient-specific anatomy rather than relying on standardized views. Complete catheter engagement and adequate contrast opacification are equally important. Each platform has specific requirements with respect to projection angle separation, frame rate, and vessel filling. Failure to adhere to these specifications may compromise the accuracy of the analysis. If images are suboptimal, repeating the acquisition at the time of angiography is preferable to proceeding with flawed data.

### 3.3. Interpretation and Adjudication

An interpretation of ADP results requires a structured decision pathway. Borrowing from QFR, values well below or above ischemic thresholds (<0.75 or >0.92) can be employed which have yielded a high sensitivity and specificity of 95% [42]. Results falling within a gray zone or obtained with marginal image quality should be interpreted cautiously and confirmed with invasive physiology. Similarly, in complex subsets such as left main bifurcation or heavily calcified disease, ADP should be considered complementary rather than definitive. A defined escalation strategy to wire-based assessment ensures that patient management is not compromised by over-reliance on software-derived physiology.

### 3.4. Training and Operator Dependence

Although marketed as automated, these systems still require operator input and oversight. Vessel contouring, landmark identification, and confirmation of adequate reconstructions are still required in all software. Training should be mandatory for all anticipated users (interventionalists, fellows, nurses, and radiology technologists) prior to deployment. This reduces both “failure rates” from poor image acquisition and mistrust in the technology when outputs do not match clinical intuition. Importantly, the operator must also recognize when ADP may be unreliable such as in heavy calcification, ostial lesions, or poor contrast filling and appropriately escalate to invasive physiology or intravascular imaging.

### 3.5. Workflow Integration

Integration is most successful when the system is built into the natural rhythm of the Cath lab. ADP analysis may be performed either on-console or via a connected workstation. Optimal integration occurs when trained staff are empowered to perform analyses during the diagnostic angiogram. This allows for real-time interpretation while the operator remains scrubbed, minimizing disruption to procedural flow. Embedding ADP into standard lab protocol with clearly assigned tasks within the team will be central to the efficient implementation of ADP software.

### 3.6. Cost and Reimbursement

Practical adoption also depends on financial considerations. Licensing and integration costs remain significant, and reimbursement pathways vary across regions. Although FDA clearance has been achieved, consistent reimbursement is not yet universal. Institutional engagement with coding and billing teams is required to secure sustainable use.

### 3.7. Regulatory and Guidelines

Clinicians must be aware of which applications are formally approved and which remain investigational. Lesion-level functional assessment is cleared for clinical use, whereas applications such as stent sizing, lesion length estimation, and residual physiology prediction remain investigational or off-label. Until confirmatory prospective evidence emerges, such features should be interpreted as investigational adjuncts rather than standard of care.

### 3.8. Post-Procedural Quality Review

Finally, institutional adoption should include a periodic review of ADP performance including correlation with invasive physiology, clinical outcomes, and rates of failed analyses. Such reviews will promote quality assurance, improve the learning curve, and can increase operator confidence over time. These reviews will also allow users to remain updated on the latest advancements with the ADP software.

## 4. Future Directions

ADP has rapidly evolved from proof-of-concept to clinical reality, obtaining regulatory approval for the assessment of intermediate lesions in patients with CAD. Large-scale clinical trials have been completed and are ongoing to further establish clinical utility compared with the current gold standard of wire-based physiology. In parallel, active research is expanding its potential applications to more complex anatomic subsets and diverse coronary pathologies, including spontaneous coronary artery dissection, cardiac allograft vasculopathy, and microvascular dysfunction. With ongoing clinical use and the accumulation of large-scale datasets, these platforms will also have the opportunity to continuously refine their performance. If trained on increasing numbers of angiograms, these algorithms may become increasingly accurate over time.

Looking ahead, one can envision ADP becoming integrated into the fluoroscopy system with results displayed in real time on the angiographic console. Future fluoroscopy systems could incorporate automated reconstruction and overlay interfaces, enabling near real-time physiologic assessment directly from cine angiography. Such advances could allow not only for rapid identification of physiologically significant lesions, but also for comprehensive PCI planning, providing stent length, vessel sizing, and predicting and confirming post-PCI physiologic effect. Once validated in prospective studies, these capabilities may transform ADP from a diagnostic adjunct into a central tool for procedural guidance and optimization.

## 5. Conclusions

ADP has emerged as a validated, wire-free method for the functional assessment of CAD with growing evidence supporting its use in routine practice. Across available platforms, ADP-guided strategies generally demonstrate superior or at least comparable performance to angiography alone, although noninferiority to wire-based FFR has not yet been established in randomized trials. Importantly, wire-based FFR remains the reference standard, requiring preservation of technical and interpretive expertise. In this context, ADP should be viewed as a complementary tool for intermediate lesions assessment, and in select cases, an appropriate alternative to wire-based FFR. While current limitations including reliance on angiographic quality, training requirements, and incomplete validation in certain populations must be acknowledged, practical solutions and ongoing technological refinement continue to address these gaps. As prospective data accumulates and integration into catheterization lab workflow advances, ADP has the potential to reshape physiologic assessment and become an integral component of contemporary PCI planning and optimization.

## Figures and Tables

**Table 1:** Comparison of angiography-derived physiology platforms.

Feature	QFR (Medis)	FFRangio (CathWorks)	vFFR (Pie Medical)
Angiographic Requirements	2 orthogonal views, ≥25° apart	≥2 orthogonal views, 30°apart	2 orthogonal views, ≥25° apart
FDA-cleared cutoff	≤0.80	≤0.80	≤0.80
Outputs	Lesion level QFR, stent sizing, post-PCI prediction (investigational)	Multilevel tree analysis, pullback, lesion impact tool, vessel sizing	Lesion-level vFFR, virtual pullback, residual vFFR (investigational)
Unique features	Microvascular assessment using IMRangio (investigational)	Whole-vessel tree map; lesion impact tool	Residual physiology prediction
Best studied in	ACS, CABG graft patency, diffuse CAD	Multivessel CAD, cost effectiveness	Left main validation (vs. IVUS), diffuse CAD

**Table 2:** Key validation studies of ADP vs. wire-based FFR.

Platform	Study	N (lesions)	Accuracy (%)	Sensitivity (%)	Specificity (%)
QFR	FAVOR II China	308	93	95	92
QFR	FAVOR II Europe-Japan	329	87	87	87
vFFR	FAST-EXTEND	294	88	75	94
vFFR	FAST II	334	90	81	95
FFRangio	FAST-FFR	301	92	94	91

**Table 3:** Randomized controlled trials of ADP.

Platform	Trial	Comparator	N	Primary Outcomes	Key Results
QFR	FAVOR III China	Angiography-guided PCI	3825	1-year MACE	QFR reduced MACE (HR 0.55), fewer PCIs
QFR	FAVOR III Europe	Wire-based FFR	2000	1-year MACE	QFR not non-inferior, more stents, increased MI
FFRangio	Provision	Wire-based FFR	401	Revascularization rate	Met non-inferiority, decreased cost and radiation
FFRangio	ALLRISE	Wire-based FFR	~1924	1-year MACE	Pending
vFFR	FAST III	Wire-based FFR	~1700	1-year MACE	Pending
vFFR	LIPISIASTRATEGY	Wire-based FFR	~2000	1-year MACE	Pending

**Table 4:** Summary of angiography-derived physiology in complex clinical scenarios.

Lesion Subsets	QFR	vFFR	FFRangio
**ACS (non-culprit lesions)**	Strong diagnostic performance vs. FFR (AUC ~0.89) with suggested triage cutoffs < 0.75 (treat) and >0.92 (defer); good agreement between acute and staged measurements	FAST-STAGED: High accuracy of acute vs. staged vFFR with (diagnostic accuracy ~94%). FAST-STEMI II: Modest diagnostic performance vs. FFR (accuracy ~71.8%) with discordance mainly in microvascular dysfunction.	NSTEMI Population: High diagnostic accuracy vs. FFR for intermediate non-culprit lesions (diagnostic accuracy ~97%)
**Left Main disease**	Excellent agreement with FFR in intermediate lesions (diagnostic accuracy ~91%)	vFFR correlates strongly with IVUS-derived MLA; vFFR ≤ 0.80 correlates well with MLA < 6.0 mm^2^	Limited: LM and ostial lesions were largely excluded from early validation
**CABG/Graft patency**	Pre-operative QFR > 0.80 is associated with increased graft occlusion; pre-op LAD QFR > 0.80 independently associated with internal mammary graft failure and adverse outcomes increased graft failure risk	Limited: No dedicated graft outcome studies.	Limited: No dedicated graft outcomes studies.
**Bifurcation lesions**	Post-PCI QFR in main and side branches identifies functionally incomplete revascularization and predicts higher long-term adverse	Bifurcation subsets within FAST-EXTENDED and FAST II show good diagnostic agreement with FFR	Limited data
**Diffuse/serial disease**	QFR pullback mimics pressure-wire pullback	vFFR incorporates press-drop analysis to evaluate individual lesions in serial disease	Limited: Whole tree-mapping highlights segments in diffuse/serial disease
**Chronic Total Occlusion**	Lower post-PCI QFR values after CTO intervention associated with worse clinical outcomes	Limited	Limited
**Severe AS**	Good agreement with FFR (diagnostic accuracy ~84%); Post-TAVI QFR maintains good agreement (diagnostic accuracy ~83%)	Limited	Limited
**Microvascular dysfunction**	QFR-derived IMRangio provides an angiography-based estimate of microcirculatory resistance showing early validation vs. wire-based IMR	Limited	Limited
**Post-PCI assessment**	HAWKEYE: Post-PCI QFR ≤ 0.89 is associated with a ~3-fold higher vessel-oriented composite events	Validated against FFR to detect suboptimal post-PCI physiology; lower vFFR associated with higher target failure at long-term follow-up	Limited
**Intravascular Imaging**	Lower QFR associated with IVUS/OCT high-risk plaque features, smaller lumen dimensions, and greater stenosis severity	Reduced vFFR correlates with adverse OCT-derived lumen metrics and high-risk characteristics	Limited: Early data shows reasonable agreement between FFRangio derived vessel sizing and IVUS measurements

QFR = quantitative flow ratio; vFFR = vessel fractional flow reserve; FFRangio = angiography-derived FFR; ACS = acute coronary syndrome; LM = left main; CABG = coronary artery bypass grafting; CTO = chronic total occlusion; AS = aortic stenosis; TAVI = Transcatheter aortic valve implantation; MLA = minimum lumen area; IMR = index of microcirculatory resistance; AUC = area under the curve; IVUS = Intravascular ultrasound; OCT = optical coherence tomography.

## Data Availability

Not applicable.

## Abbreviations

The following abbreviations are used in this manuscript

ACS	Acute Coronary Syndrome
ADP	Angiography-Derived Physiology
AS	Aortic Stenosis
CABG	Coronary Artery Bypass Grafts
CAD	Coronary Artery Disease
CFR	Coronary Flow
CTO	Chronic Total Occlusion
FFR	Fractional Flow Reserve
FDA	U.S. Food And Drug Administration
IVI	Intravascular Imaging
IVUS	Intravascular Ultrasound
LM	Left Main
MACE	Major Adverse Cardiac Events
MI	Myocardial Infarction
MLA	Minimal Lumen Area
NHPR	Non-Hyperemic Pressure Ratio
NPV	Negative Predictive Value
OCT	Optical Coherence Tomography
PCI	Percutaneous Coronary Intervention
PPV	Positive Predictive Value
QFR	Quantitative Flow Ratio
STEMI	ST Elevation Myocardial Infarction
TAVI	Transcatheter Aortic Valve Implantation
vFFR	Vessel FFR
